# Experiences of people with diabetes receiving a voucher for healthy foods: a qualitative study

**DOI:** 10.1186/s12889-026-26436-y

**Published:** 2026-02-10

**Authors:** Moizza Zia Ul Haq, Lavanya Sinha, Chinwe Nwebube, Adelaide Buadu, Areesha Sabir, Nav Persaud

**Affiliations:** 1 MAP Centre for Urban Health Solutions, Li Ka Shing Knowledge Institute, St. Michael’s Hospital, Unity Health Toronto, 209 Victoria St, Toronto, ON M5B 1T8 Canada; 2 Department of Family and Community Medicine, University of Toronto, 500 University Ave, 80 Bond St., Toronto, ON M5B 1X2 Canada

**Keywords:** Food prescription, Diabetes, Food insecurity, Food voucher

## Abstract

**Background:**

Food insecurity is linked to lower consumption of healthy foods in people with diabetes. Food prescription programs may improve diabetes outcomes and promote fruit and vegetable intake among people with diabetes. We conducted a qualitative study to understand the experiences of people with diabetes or prediabetes facing food insecurity who were receiving a monthly food voucher as part of a clinical trial.

**Methods:**

For this qualitative study, we purposefully selected a subset of participants in a clinical trial who were randomized to receive a monthly $65 Canadian voucher for healthy foods for six months. Participants were adult primary care patients with diabetes or prediabetes who also reported food insecurity or trouble making ends meet. We conducted semi-structured interviews and analysed interview transcriptions using an inductive approach to derive themes.

**Results:**

We interviewed 20 participants. Many participants reported an awareness of how to eat in accordance with diabetes-management and the vouchers provided participants the freedom able to eat in such a way, allowing them to purchase healthier foods without having to worry about finances. The vouchers also gave participants more agency over their health and had positive impacts on their mental and emotional well-being. A few participants reported feeling embarrassed to use the voucher. The voucher amount was insufficient for many participants. They described several ways to make the most effective use of the vouchers, including purchasing items on sale.

**Conclusion:**

The experiences shared by the study participants suggest that a monthly voucher for healthy foods may improve well-being by providing freedom to eat foods they know help to improve health and manage diabetes.

**Trial registration:**

This article is linked to the Vouch 4 Diabetes randomized controlled trial (Trial Registration at ClinicalTrials.gov NCT05776420, registered Mar 16, 2023).

**Supplementary Information:**

The online version contains supplementary material available at 10.1186/s12889-026-26436-y.

## Background

Food insecurity affects 23% of people in Canada or 9 million people according to 2022 estimates, and this number is growing [1]. Food insecurity is associated with increased risk for type 2 diabetes [2], as well as poor glycemic control and diabetes distress for those already diagnosed [3–5]. Individuals with a low income who have diabetes or prediabetes are less likely to adhere to dietary management guidelines and are at an increased risk of developing complications [6–8]. Food insecurity is correlated with reduced diet quality and lower consumption of healthy foods in adults with diabetes [9]. Individuals living with type 2 diabetes and facing food insecurity have difficulty affording enough food, and difficulty affording healthier, high-quality foods. Many describe having to stretch or skip meals or choose foods considered to be unhealthy because they are less expensive [10, 11]. People are forced to choose between purchasing healthy foods or being able to afford necessities, such as medications or rent [11, 12].

Food prescription programs, where patients are provided vouchers for or boxes of healthy foods in a health care setting, have garnered interest as a potential way to alleviate food insecurity and current literature shows that food prescription programs are beneficial. One scoping review demonstrated that this type of program may improve fruit and vegetable consumption and reduce food insecurity [13]. Some studies demonstrate that food prescription programs reduced HbA1c levels amongst participants with type 2 diabetes [14, 15]. This is especially true for food prescription programs that encouraged participants to increase fruit and vegetable intake [16]. However, certain studies have only demonstrated short-term improvements in health outcomes [15]. These positive outcomes were not sustained past the duration of the intervention. It is important to examine the benefits of such food prescription programs, as well as understand and address barriers to uptake and access. Social perceptions and judgement from others can especially impact participants’ willingness to use such interventions. For example, there may be concerns from some participants about stigma around voucher use [16].

This qualitative study was embedded within a randomized controlled trial (RCT) with 390 participants that aimed to measure the effect of a monthly voucher that allows primary care patients with diabetes or prediabetes to access healthy foods. The purpose of this study was to understand the experiences of participants randomly allocated to receive a monthly subsidy of $65 Canadian (or $85 for participants who were members of households with more than five individuals) for fruits and vegetables every month for six months, in the form of a gift card redeemable at grocery retail stores. Participants received a handout that included advice about healthy eating with diabetes in addition to usual care that involves meeting with a nurse or dietician to discuss diet and exercise. Quantitative results of the trial indicated the voucher slightly improved glycemic control although the results were not statistically significant and there was a substantial reduction among people with prediabetes [17]. The results of this qualitative study will help to understand the settings in which the findings of the RCT may or may not be applicable, and highlight potential harms or barriers associated with food voucher use.

## Methods

This was a qualitative study of participants receiving the intervention in a clinical trial examining the effects of a $65 monthly healthy food voucher. The trial and this qualitative study were conducted in Ontario, Canada where there is no governmental assistance related to healthy foods but where social assistance recipients receive a financial supplement for healthy foods if they have diabetes. The value of the voucher in the study was similar in size to this supplement. The voucher could be used to purchase anything sold by a national grocery chain selected by each participant. Participants in the control arm of the RCT received usual clinical care. We used a generic qualitative framework for this study [18]. More specifically, an interpretive description methodology was used, because it allows for the consideration of study participants’ attitudes, thoughts, and experiences to generate practical findings [19].

### Participants and sampling

Participants in the qualitative sub study were selected from the main randomized controlled trial. The RCT included 390 adult (19 years or older) primary care patients with diabetes or prediabetes who also reported food insecurity or trouble making ends meet using validated instruments [17]. The seven trial sites were: The Health Centre at 80 Bond, 61 Queen Family Practice Unit, Wellesley – St. James Town Health Centre, Sumac Creek Health Centre, St. Lawrence Health Centre, Regent Park Community Health Centre and South Riverdale Community Health Centre. All enrolled participants provided written informed consent.

Participants in the intervention arm of the trial who consented at baseline to being contacted for the qualitative study were invited via phone to participate in a 20-minute interview. Twenty participants in the intervention arm of the study were purposefully selected for the qualitative sub study. Participants with a variety of genders, household incomes, household sizes, and conditions (i.e. diabetes or prediabetes) were selected. Participants were interviewed while receiving the intervention or after completing trial follow-up. Participants were compensated with a $20 grocery gift card.

### Data collection

The interview questions were developed with the community guidance panel that was set up for a randomized controlled trial of access to free medicines [20]. The questions for this qualitative study were adapted from questions used in the previous trial of medicine access qualitative concept mapping study, based on input from the community guidance panel [21]. We conducted semi-structured interviews with twenty participants over the phone. Our interview guide consisted of 10 questions that aimed to understand the participants’ experiences with the voucher (see Additional file 1, Appendix 1). The questions aimed to understand how the voucher was used, potential benefits and downsides of use, and any barriers encountered during the study. Two research team members (A.S. and M.U.) conducted interviews over the phone between January 2024 and November 2024. The interviewers stuck closely to the interview guide and follow-up questions were asked for clarification. The first three interviews were not recorded. After receiving approval from the ethics board for audio recording of interviews, the remaining 17 interviews were audio recorded on an audio recording device, upon receiving verbal consent from participants. The research assistants took notes during each interview. The interviews were subsequently transcribed using the Otter.ai software (Otter.ai Web version 3.69.1) and de-identified. After each interview, the research team met to discuss any preliminary findings. While a purposeful sample of 20 participants was originally selected, the research team was prepared to conduct further interviews if data saturation was not yet achieved. However, after data collection from 20 interviews, saturation was achieved, as no new substantive findings were obtained.

### Data analysis

De-identified transcripts were uploaded onto the ATLAS.ti software (ATLAS.ti Web, version 9.11.4) for qualitative analysis. The interview transcripts were analysed on the ATLAS.ti software and manually coded using an inductive approach. Each transcript was coded independently by two researchers (M.U. and L.S.). Both researchers read through each transcript and added codes that were guided by the data. For each transcript, the two coders met to discuss the initial coding and resolve any disagreements. The codes were then reviewed and discussed by the two coders (M.U. and L.S.) and another research team member (C.N.), and, in an iterative process, grouped together based on commonalities in subjects and experiences raised by participants. Through further discussions between the research team members, the code groupings were clustered together into overarching themes that summarized the data and sub-themes that captured the variety of participants’ experiences while accurately representing the initial codes. The research team met on a weekly basis to review and discuss emerging concepts and themes.

## Results

We approached 20 purposefully selected participants, and all 20 agreed to participate. We conducted 20 interviews that lasted between approximately 4 and 29 min. The sample included 12 women (60%) and 8 men (40%) with either diabetes (*n* = 14, 70%) or prediabetes (*n* = 6, 30%). The participants’ yearly household incomes ranged from less than $5000 to $70 000 and 7 (35%) were social assistance recipients, with household sizes ranging from 1 to 6 people. The participants were from varied racial and ethnic backgrounds including those who identified as Black (*n* = 6), South Asian (*n* = 4), Latin American (*n* = 3), Arab (*n* = 1), White (*n* = 1), Chinese (*n* = 1), Japanese (*n* = 1), West Asian (*n* = 1), and Other (*n* = 2, which included Mixed or Indo-Caribbean). Most (*n* = 17) were interviewed after completion of their 6-month follow-up, and 3 participants were interviewed after receiving 2 or 3 monthly vouchers. Our themes and representative quotes are summarized in Additional file 1, Appendix 2.

### Uses of the voucher

Participants reported mainly using the voucher to purchase fruits and vegetables, which could include fresh and frozen produce. One participant described purchasing organic fruits and vegetables, in particular. While some participants used the voucher only for fruits and vegetables, others shared that they purchased other foods in addition to fruits and vegetables with the voucher. These included dairy products, poultry products, breads and grains, tea, and bottled water. Many participants described that they shared the groceries purchased through the voucher with members of their household, including their partners or family members. A few participants mentioned that their family members did not like fruits and vegetables so they did not share the voucher with their family. Only two participants mentioned occasionally sharing the voucher with individuals outside their household.

### Direct benefits of the voucher

Many participants described having a strong understanding of how to eat in accordance with diabetes management guidelines or knew the importance of eating fruits and vegetables and so used the voucher to shop and eat in ways that reflected this knowledge. Many participants expressed that healthy foods, in particular, had become increasingly expensive. Some described being unable to buy healthy foods or being forced to buy unhealthy foods due to financial constraints prior to receiving the voucher.

The voucher provided participants the ability to buy the foods they knew to be healthy for them, but could normally not afford. Participants valued the ability to purchase foods they wanted without worrying about finances.

*“I was able to buy fruits and vegetables freely for the first time. In the past*,* I just bought foods would fill me up like pasta*,* rice*,* things like that.” – 1*.

Some explained that the voucher allowed them to be more conscious of what they were purchasing, enabling them to buy fruits and vegetables over other, less nutritious foods. They could purchase more groceries and were able to incorporate more fruits and vegetables into their diet and lifestyle.

*“It brought to my awareness what I was procuring*,* what I was buying*,* and I focused more on fruits and vegetables instead of carb intensive products.” – 5*.

Some found the pamphlet that was provided with the voucher to be helpful as a reminder and guide for what foods they should eat.

On the other hand, despite their knowledge regarding healthy eating, a few participants continued to purchase and eat foods that could negatively affect their diabetes. They mentioned that additional education such as a “mini primer” (Participant 3) around appropriate foods and serving sizes would have been helpful.

Furthermore, the voucher helped participants purchase a greater variety, or purchase better quality fruits and vegetables. One participant described that with the voucher, they felt more comfortable trying fruits and vegetables that they would not normally eat.

*“Before there was only banana and apple only in my home*,* but [now] I can buy mango*,* strawberry*,* different fruits.” – 13*.

### Indirect benefits of the voucher

Use of the voucher also provided many participants with a sense of agency, as the extra funds from the voucher helped participants feel more in control of their food choices. One participant noted that the ability to afford healthy foods made them feel significantly better about their health, as they felt control over what they were putting in their body. Another participant mentioned losing weight because of the better food choices they were making. Knowing they had extra funds to spend on healthy foods motivated participants to take care of their health.

Some participants reported that the freedom to buy the foods they wanted positively impacted their mental or emotional well-being. One participant noted the voucher improved their mental well-being which ultimately facilitated a reduction in their substance use, a “ripple effect” beyond diet. Participants also experienced positive emotions from the voucher, particularly happiness and pride from having the freedom to purchase groceries that they wanted and liked. One participant described that because they were able to save money on groceries with the voucher, they had funds to engage in enjoyable activities that improved their well-being.

A few participants described how the benefits of the voucher extended to their children. One participant reported that her children enjoyed having more fruits and vegetables, and two reported that they bought treats for their children either directly with the voucher or with the money saved from using the voucher.

*“I have three children*,* and although*,* like when they came*,* I was able to buy things like birthday cake and*,* you know*,* the extra stuff*,* yeah*,* I didn’t put it on the card*,* but I had extra income*,* where… I had the resources to do these meaningful things.” – 5*.

In addition, a benefit that participants, particularly in the lowest income bracket, described was alleviation of their food insecurity. Prior to the study, financial constraints led some participants to make use of food banks, eat meals in a shelter, or eat only one simple meal a day. With the voucher, participants noted having extra money to buy what they needed to supplement the meals they received at the shelter or food banks. In addition, they had enough food to eat multiple meals a day. One participant mentioned that they appreciated the voucher because they could avoid taking food bank meals from those they considered in more dire need. For others, the voucher allotted extra money to spend on groceries due to an otherwise limited budget, or provided a supplement to their income, particularly when in between paychecks or on a limited income.

A few participants reported that the voucher helped them make ends meet, as the voucher helped offset costs for other necessary expenses such as rent and bills. Money was saved for other expenses, because participants spent less of their own money on groceries.

*“So my pension is paying towards my rent*,* and so my monthly grocery*,* you know*,* I have to be very*,* very careful how to how to use*,* how to spend it. Sixty dollars*,* it helps a lot*,* because instead of*,* I will be spending*,* let’s say*,* $150 or*,* you know*,* $200 a month [on] grocery*,* you know*,* you minus that $60 so*,* you know*,* it helps a lot. I would just have to dish out maybe 120*,* $140 of my own allowance.” – 6*.

### Drawbacks or harms of the voucher

#### Insufficient amount

A common theme amongst participants was that though the gift card was useful, it was insufficient to support their grocery needs. This viewpoint was expressed often by participants in multiple-person households, but also by individuals who lived alone. Many expressed that the voucher amount did not reflect the high prices of groceries.

*“No*,* $60 is not enough to share. No*,* no*,* it’s not enough. And*,* yeah*,* I live by myself in my in the apartment. So that $60 it helps. Sometimes it doesn’t. It’s not enough because you know how expensive [groceries] are.” – 6*.

When the voucher ran out, participants would have to spend their own money to pay for the remainder of their groceries. One participant described being embarrassed if the card ran out and they were left unable to pay for their groceries.

*“Sometimes when the card ran out of balance*,* and I couldn’t afford the groceries*,* that was embarrassing.” – 2*.

Many participants described how they made most efficient use of the value of the voucher. They shared that they were mindful of the available funds on the voucher to avoid overspending or running out of funds during the month. One participant described waiting for their reload before going grocery shopping.

One participant explained that they saved the voucher for when they were between paychecks. Another considered the voucher while budgeting for other expenses to make sure it was being used effectively. Participants highlighted that they continued to purchase things on sale or purchase cost-effective items to maximize the value of the voucher. One participant even collected loyalty points which could then be redeemed towards discounts on future purchases.

Participants also employed various strategies to ensure the food they bought with the voucher lasted as long as possible. Some described planning their meals or rationing their groceries to make them last across multiple meals or days. Others bought certain fruits or vegetables that were likely to last, such as frozen or in-season produce. One participant described preparing fruits in ways to make them last longer, such as incorporating them in salads or juices.

However, one drawback of the voucher was the lack of flexibility around the grocery stores where the voucher was eligible. A few participants mentioned that despite having the voucher, they used their own money to buy fruits and vegetables at alternative, non-voucher affiliated grocery stores, such as the local Chinese grocery store. This was because the produce there was less expensive, better quality or more readily available. A few participants noted that non-voucher grocery stores had better sales or they made use of discount food service apps. As such, the ability to use the voucher at other grocery stores or chains would help the participant shop in a more cost-effective way. Some other participants mentioned that they could not use the voucher at their “usual” grocery store or chain, and going to a voucher-eligible store was a hassle. Some participants went to both voucher and non-voucher affiliated grocery stores to obtain groceries.

#### Barriers

Some participants experienced logistical barriers with the voucher. For instance, one participant would forget the voucher at home and have to use their own money to pay for their groceries. Another participant had to rely on their partner’s availability to drive them to the grocery store and back. A few participants mentioned that the groceries were sometimes too heavy to carry home. One individual had to make multiple trips to transport all of their groceries. Lastly, one participant found that putting the fruits and vegetables on a separate transaction each time felt awkward.

Despite some of the stigma and logistical difficulties associated with use of the voucher, having access to money to purchase fruits and vegetables had a variety of positive outcomes including increased access to healthy foods and increased agency over their food purchases. However, according to some participants, these benefits did not extend past the cessation of the study. Once the study ended, the participants no longer had access to the grocery voucher and therefore could not maintain their purchasing habits as seen in the study. As such, a limitation to the voucher as expressed by participants was that it only lasted for 6 months.

#### Stigma

Many participants shared that they did not experience stigma with the use of the voucher. They themselves were not ashamed of using it, and did not experience negative comments from individuals outside of the study. In fact, the gift card format seemed to mitigate negative stigma often associated with use of the Supplemental Nutrition Assistance Program (SNAP) because it was a very common form of payment that did not stand out as a subsidy.

One participant expressed gratitude for the gift card format, as handling cash was a trigger for them in the context of substance use.

On the contrary, there were some participants that did feel embarrassed using the voucher, for reasons including that it made them feel othered compared to other shoppers.

*“It’s a little embarrassing. People don’t necessarily know where it came from. It just makes me feel like the other*,* if I’m not tapping a card like everyone else is right?” – 16*.

For one individual, they did not feel embarrassed using the voucher, but their partner did.

## Discussion

This qualitative study of the experiences of clinical trial participants with diabetes and prediabetes found that a voucher for healthy foods supported freedom to purchase healthier foods without having to worry about finances, gave participants more agency over their health, and positively impacted mental well-being. Most participants expressed concern at the rising prices of healthy foods and participants used different techniques, such as rationing their groceries, to make the voucher last. The two most reported downsides to the voucher were that the amount was insufficient, and that there was a lack of flexibility around which grocery stores the voucher could be used.

Our findings align with other studies of interventions aimed to increased financial access to fruits and vegetables. In studies of food prescription programs in the United States, participants reported that the program provided economic support to help increase and improve the quality and variety of their fruit and vegetable consumption, especially since healthy foods were often out of reach [16, 22–25]. Like our study, others have found that participants had an understanding of healthy eating, but financial constraints and high costs of groceries limited their ability to purchase healthy food, which was found in studies of food support programs [22, 24], and studies examining barriers to healthy foods among low-income or food-insecure populations [26–28]. Diabetes self-management education is emphasized in the Canadian Diabetes Guidelines [29] and people with diabetes in Canada recognize the importance of gaining knowledge about their condition and lifestyle interventions to manage their condition [30]. However, research suggests that although improving nutritional knowledge may be helpful in addressing health and diabetes outcomes, this alone cannot address disparities faced by people with severe food insecurity [31].

A qualitative study of a food prescription program in Ontario found that the program enabled participants to extend their government-funded income supports and divert money they would have used to buy food towards other necessities [32]. Similarly, participants in our study reported that the voucher provided a supplement to their income, including for those that received government subsidies such as the Ontario Disability Support Program.

A key barrier highlighted in other studies of grocery incentive programs was transport to and from the grocery store [16, 22, 24], however, transportation as a barrier was only expressed by a few participants in our study. This may be because our participants were able to select a voucher redeemable at a grocery chain that was closer to them. In addition, our study was conducted in an urban setting with access to various modes of public transport (i.e. bus, subway, streetcar) which perhaps mitigated the need for a car as the primary mode of transportation. Stigma was identified as a barrier in other similar studies [24, 33]. Our findings indicated that a few participants felt embarrassed to use the voucher, while others did not because the gift card format was not perceived as a subsidy. Another barrier highlighted in similar studies was that participants were limited to the purchasing “fresh” produce either in grocery stores, or farmers markets [22, 34]. This resulted in participants being unable to buy fruits and vegetables in other forms. Our study allowed participants to purchase varied forms of produce, including cheaper items and longer-lasting items (i.e. frozen or canned) which participants cited as a modality to extend the voucher use.

Some other studies have noted that there were limits in how much could be purchased with the grocery incentive [22] and participants suggested increasing the amount provided [35]. Our study explicitly asked whether the voucher was sufficient, and participants overwhelmingly expressed that voucher amount was felt to be insufficient to support their grocery needs. Finally, participants in a study of a produce prescription program found, similar to our study, that patients were not able to sustain the improved eating habits after the program [22]. This highlights the need to study and address upstream drivers of food insecurity and rising food prices to ensure meaningful and sustainable improvements in health outcomes. While food voucher programs may decrease food insecurity during the duration of the interventions [36], research suggests that comprehensive public policies that alleviate poverty are crucial [37].

### Strengths and limitations

A key strength of this study was that we included a diverse subset of participants in terms of household incomes, household sizes, and conditions, which allowed us to capture a wide array of participant experiences. Having two researchers code transcripts and meeting regularly to discuss the interpretation of the findings increased reliability and trustworthiness of our results. Three interviews were not audio recorded. Experiences with longer-term use of a voucher could not be assessed in this six-month study. Experiences would likely be different with vouchers that are greater (or even smaller) in value compared with the voucher used in our study. The findings from our study conducted in a large city in the Global North would not necessarily apply to other settings. Finally, this was a qualitative study, so quantitative measures of the impacts of the voucher, such as effects on hemoglobin A1c and fruit and vegetable consumption were not analysed – these results of our trial are reported separately.

## Conclusion

People living with type 2 diabetes or prediabetes facing food insecurity found a $65 monthly grocery voucher to beneficial, as it allowed them the freedom to eat foods that they already knew would improve their health, well-being, and management of diabetes. The voucher amount was insufficient for many participants, and a key drawback was the lack of flexibility in which stores they could use the voucher. Future work may address the effects of larger vouchers provided for longer durations.

## Supplementary Information

Supplementary Material 1.

## Data Availability

The data are available from the corresponding author on reasonable request.
