# Supplementary material for: Experiences of people with diabetes receiving a voucher for healthy foods: a qualitative study
Source: BMC Public Health. 2026 Feb 10;26:892. doi: 10.1186/s12889-026-26436-y (PMC12990521; doi:10.1186/s12889-026-26436-y)
Supplement: Supplementary file 1 — Supplementary Material 1. [file 12889_2026_26436_MOESM1_ESM.pdf]

Appendix 1. Qualitative Interview Guide

Thank you for agreeing to be part of this sub-study.

I will be asking you a few questions about your experiences with the food voucher. Please answer as accurately as you can. Also, please feel free to ask me if you need any clarifications.

This information will be kept confidential and any data used for research purposes will not link you to any of the information you provide.

Please feel free to stop me at any time if you no longer wish to continue the interview.

With your consent, this interview will be audio recorded. However, all recordings will be deleted after I have had a chance to take notes. Do you consent to being audio recorded during this interview? (Yes) / (No)

1. Overall, what was your experience using the vouchers to obtain food at the supermarket? [prompts can include: did you experience any barriers? Any issues having them accepted? Forgetting them at home? Was it a positive/negative experience?]

2. Could you discuss the benefits of the vouchers, if any?

3. Could you discuss the downsides of the vouchers, if any?

4. What did you use the vouchers for?

5. Did you share the vouchers (or the purchased items) with others in your household?

6. Did you share the vouchers (or the purchased items) with others outside of your household?

7. Did you have trouble getting to the supermarket or getting home with your groceries?

8. Were you ever embarrassed or ashamed of using the vouchers? Did anyone ever say anything negative about you using the vouchers?

9. Do you feel the monthly voucher was sufficient to cover the cost of a month’s supply of fruits and vegetables for you or your family?

10. If not already discussed: How did receiving the vouchers effect your ability to make ends meet at the end of the month?

Appendix 2. Themes and representative quotes

Theme	Representative Quotes
Uses of the voucher	
Participants reported mainly using the voucher to purchase fruits and vegetables.	I use[d] [the voucher] for vegetables, and fruits, too…. I used it to buy, cabbage, carrots and broccoli, cauliflower, tomatoes…zucchini. Yes, that was very good for me." – 11
Some participants purchased other foods in addition to fruits and vegetables with the voucher (e.g. dairy products, poultry products, breads and grains, tea, bottled water).	"Sometimes for dinner, I buy a bread. And that bread I make it go for like, five or seven days, because I will use, like, one slice" – 15<br><br>"I just buy whatever that that I that benefit me… for most of the $[65] I use it on, on meat or fish or banana, fruits. it helped a lot." – 6
Direct benefits of the voucher	
Participants used the voucher to shop and eat in ways that reflected their knowledge of how to eat in accordance with diabetes management guidelines.	"I ate things that were healthy and food from the ground, instead of bleached out and processed food." – 1
Many participants expressed that healthy foods were expensive. Prior to the voucher, some were unable to buy healthy foods or forced to buy unhealthy foods.	"Everything is expensive, so when you go to the grocery store, you don't think about fruits and vegetables." – 14<br><br>"I'm used to buying food on the road, that wasn't good for me, it raised my blood sugar. I had to eat what I'm not supposed to eat."– 11
Participants valued the ability to purchase foods they wanted without worrying about finances.	"For me, every time I go to the supermarket, I'm always like, I'll buy what [my family] will eat…. My husband and my son, they don't eat fruit. But [with the voucher] I got the chance to buy all the fruits that I wanted to eat." – 14<br><br>"Just having the voucher gets me into a mode of thinking that like, like, I will reach for fruits and veggies more, because I know I have that financial help." – 16<br><br>"I was able to buy fruits and vegetables freely for the first time. In the past, I just bought foods would fill me up like pasta, rice, things like that." – 1
The voucher allowed participants to be more conscious of what they were purchasing, enabling them to buy fruits and vegetables over other, less nutritious foods.	"[The voucher]'s gotten me thinking more about fruits and vegetables. I know that I have that extra financial help. It's got me thinking about different ways I can incorporate them into my diet, but not just having them on hand, but like different cooking methods." – 16<br><br>"It brought to my awareness what I was procuring, what I was buying, and I focused more on fruits and vegetables instead of carb intensive products." – 5
A few participants continued to purchase and eat foods that could negatively impact their diabetes.	"I didn't do as good my expectation, because during summer time, I don't really care so much about, like, my condition I just got sugary fruits and sweets, which is not allowed for me to have, but I couldn't control" – 7
The voucher helped participants purchase a greater variety, or purchase better quality fruits and vegetables.	"Instead of buying corn, like regular corn, I would buy the frozen one or the can one, right? Because, because the regular one, is too expensive. So then now, because of the card… we can have a better, a better quality, instead of buying like the green beans in the can, we buy regular green beans, right, or regular corn." – 20

	"Before there was only banana and apple only in my home, but [now] I can buy mango, strawberry, different fruits." – 13
Indirect Benefits of the Voucher	
The voucher provided participants with a sense of agency over health or food choices.	"It did make a huge difference - it helped me feel more active, more in control, and better about my health." – 1
The freedom to buy the foods they wanted positively impacted participants' mental or emotional well-being.	"As an example, I had a little bit extra cash, so I was able to go out to dinner with my friend, and helping my socialization, because I had a little bit extra…. The socialization, enabling social contact in that, you know, going out for a meal or maybe doing something together, like a fun activity, or something like that,... I attribute that to the grocery card program." – 5
Benefits of the voucher extended to participants' children.	"I have three children, and although, like when they came, I was able to buy things like birthday cake and, you know, the extra stuff, yeah, I didn't put it on the card, but I had extra income, where… I had the resources to do these meaningful things." – 5
Voucher alleviated food insecurity or helped make ends meet.	"Some days before I had a card or like now, I can't afford to have a breakfast, so I have a cup of tea and I have a bagel. I leave that to have for my lunch. So when I had the card, I could have had breakfast and I could have something for lunch, and maybe sometimes, what I make, I try to leave something that I could have some things to eat for dinner." – 15<br><br>"So my pension is paying towards my rent, and so my monthly grocery, you know, I have to be very, very careful how to how to use, how to spend it. Sixty dollars, it helps a lot, because instead of, I will be spending, let's say, \$150 or, you know, \$200 a month [on] grocery, you know, you minus that \$60 so, you know, it helps a lot. I would just have to dish out maybe \$100 or \$120, \$140 of my own allowance." – 6
Drawbacks or harms of the voucher	
Many participants did not experience stigma with the use of the voucher.	"No, I realized the food voucher was just a common gift card and so no one could tell that it was a subsidy or social assistance. There was no stigma because it just looked like a common gift card, and they also sell those gift cards at the store, so I did not feel ashamed using the card" – 1
Though the voucher was useful, it was insufficient to support participants' grocery needs.	"Not really enough, but something is better than nothing, I would say. Not really enough, we are four people, kids and adults. And things is now expensive as you know. So it's not really enough supported. But as I go at least something is better than nothing. So if I have \$100 bills, maybe I can \$20 or \$30 save" – 7<br><br>"No, there was no problem, obviously, between higher amounts, but it helps anyway……. We are six members, so we spend a lot." – 8<br><br>"And negative, not necessarily, but the card, it's really highlighted, like how expensive healthy eating is because it does help, right, like it helps me get a couple more things than I would have gotten, but to sustain, like an actual full produce focused, like meal plan does not seem doable for me." – 16

	"No, $60 is not enough to share. No, no, it's not enough. And, yeah, I live by myself in my in the apartment. So that $60 it helps. Sometimes it doesn't. It's not enough because you know how expensive [groceries] are." – 6
Participants had to spend their own money to pay for remainder of groceries when voucher ran out.	"Yeah, it's not enough on your card, so you have to pay $4 or $5 or $6 extra." – 6<br><br>"You know, I was always a bit surprised when I thought, you know, I'd ring up the groceries and I had to supplement it because the gift card had run out." – 18<br><br>"Sometimes when the card ran out of balance, and I couldn't afford the groceries, that was embarrassing." – 2
Participants employed strategies to make most efficient use of the value of the voucher.	"On the bill, it states how much money you have left. So next time, when I go to purchase, I know in what range I have to pay, which is important for me, because I know my limits with it." – 11<br><br>"It was $65 every month, so I tried to buy things that were on sale. So being thrifty and making it last." – 5
A drawback to the voucher was the lack of flexibility around the grocery chains where the voucher was eligible.	"Even with the card, I do a little bit of the price check. Then I see that something is cheaper [at an alternative grocery chain], so I go there to buy in the [alternative grocery chain] instead. But it's not with the card because the card don't allow me to buy there." – 20
Barriers	
Some participants experienced logistical barriers with the voucher (e.g., forgetting the voucher at home, carrying groceries home).	"Usually she goes to grocery store, my wife, with me sometimes. Sometimes she says, okay, I'm gonna go pick [the groceries] up. It's hard, or sometimes heavy." – 13
The benefits of the voucher did not extend past the cessation of the study.	"So maybe, like, if I were to say something bad about the voucher, would be it is only for six months." – 20<br><br>"Once the voucher was over, I went back to my old ways. I was like, okay, I don't have this extra money, so I'll just go back to buying whatever……. For six months, I really enjoyed fruits to the point where I started craving it again. Yeah, yeah. I kind of miss having it and again. It's just the fact that you have that gift card and then go and get it. Then you go back to your normal way." – 14
